# Regulation of Müller glial reprogramming by the stress response protein REDD1 and its limitations in preventing retinal degeneration

**DOI:** 10.1016/j.jbc.2026.113583

**Published:** 2026-09-23

**Authors:** Sandeep M. Subrahmanian, Siddharth Sunilkumar, Shreshty Budakoti, Esma I. Yerlikaya, Allyson L. Toro, Alistair J. Barber, Michael D. Dennis

**Affiliations:** 1 Department of Cell and Biological Systems, Penn State College of Medicine, Hershey, Pennsylvania, USA; 2 Department of Ophthalmology, Penn State College of Medicine, Hershey, Pennsylvania, USA

**Keywords:** DDIT4, retinal degeneration, age-related macular degeneration, glycogen synthase kinase (GSK-3), transforming growth factor beta (TGF-β), SMAD transcription factor

## Abstract

Studies here investigated the role of the stress-response protein regulated in development and DNA damage response 1 (REDD1) in Müller glial reprogramming in response to sodium iodate (NaIO_3_)-induced retinal degeneration. We previously demonstrated that global REDD1 deletion protected mice from NaIO_3_-induced retinal damage. NaIO_3_ promoted Müller glial remodeling, characterized by increased levels of glial fibrillary acidic protein (GFAP) and the horizontal orientation of Müller glial processes that localized to areas of retinal degeneration. NaIO_3_ increased TGF-β1 and mesenchymal gene expression in the retina, including upregulation of N-Cadherin in Müller glia. Retinal gliosis and mesenchymal markers were reduced in the retina of REDD1-deficient mice as compared to wild-type mice after NaIO_3_ administration, but this was likely secondary to retinal protection. TGF-β signaling was also investigated in human MIO-M1 Müller cell cultures. TGF-β1 increased REDD1 and the expression of gliosis and mesenchymal markers in wild-type Müller cells, and REDD1 deletion reduced TGF-β1-induced mesenchymal marker expression. REDD1 and its downstream effector GSK3β were required for TGF-β1-induced Smad2 linker domain phosphorylation and mesenchymal marker expression. To investigate the role of REDD1-dependent Müller glial reprogramming in retinal protection, NaIO_3_ was administered to Müller-glia specific REDD1 knockout mice. Müller glia-specific REDD1 deletion attenuated Müller cell remodeling; however, NaIO_3_-induced retinal damage was similar in Müller glia-specific REDD1 knockout and floxed control mice and visual function was only modestly preserved. The findings support the role of REDD1-dependent signaling in Müller cell reprogramming and implicate a critical role for REDD1 in cells beyond Müller glia in pathological retinal degeneration.

Age-related macular degeneration (AMD) is the leading cause of blindness in the elderly, which is characterized by progressive degeneration of the retinal pigment epithelium (RPE) and the loss of photoreceptors within the macula (1). In addition to these well-characterized changes in the RPE and photoreceptors, recent studies support that retinal Müller glia may also contribute to AMD progression (2, 3, 4). Müller glia are the principal glial cells of the retina and are responsible for numerous essential functions, including providing critical metabolic support and maintenance of retinal homeostasis (5). Müller glia extend radially across the entire retina from the inner to the outer limiting membranes. In the context of geographic atrophy (GA), an advanced form of dry AMD, Müller glia activation, remodeling, and migration are observed in regions of RPE atrophy and photoreceptor loss (2, 6, 7, 8). Atrophic regions of the retina are closely associated with subretinal membranes composed of Müller glial processes (4). While this glial membrane formation may serve to fill retinal breaks and replace degenerated neurons, it also restricts neuronal migration, axonal regrowth, and endogenous retinal regeneration (9).

In response to retinal injury, Müller glia become activated and undergo adaptive changes characterized by the increased production of growth factors and cytokines (9, 10). While glial activation likely mitigates tissue damage, sustained activation of Müller cells can contribute to the development of retinal complications and is characterized by Müller cell gliosis and the loss of normal glial functions. Müller glia can acquire mesenchymal-like characteristics through upregulation of mesenchymal markers and downregulation of Müller cell-specific markers (11, 12). Transforming growth factor-β (TGF-β) is a well-known inducer of mesenchymal transition in multiple cell types and plays a central role in fibrotic disease pathology (13). Canonical TGF-β signaling is mediated by phosphorylation of the heteromeric SMAD transcription factor complex (Smad2/3/4). TGF-β type 1 receptor directly phosphorylates an *SSXS* motif within the C-terminal tail of Smad2/3 that promotes Smad4 binding, nuclear translocation of the SMAD complex, and consequently mesenchymal gene expression. In human Müller cell cultures, TGF-β1 promotes expression of mesenchymal markers, including alpha smooth muscle actin (αSMA) (12). Although the induction of mesenchymal marker expression in Müller cells has been reported in models of diabetic retinopathy (11) and other fibrogenic conditions (12), the molecular mechanisms that regulate this response in the context of AMD have not been previously explored.

Proof-of-concept studies support that the stress-response protein regulated in development and DNA damage response 1 (REDD1; also known as DDIT4/RTP801) contributes to retinal pathology in AMD (14). We recently demonstrated that REDD1 protein abundance is increased in both the retina and RPE of mice with sodium iodate (NaIO_3_)-induced retinal degeneration (15). Mice administered NaIO_3_ are a helpful experimental model, as they mirror many of the clinical and histologic features of the expanding GA in dry AMD, but within a dramatically shortened timeframe. NaIO_3_ induces primary RPE damage and macrophage accumulation, resulting in photoreceptor injury and thinning of the outer and inner segments. Remarkably, whole-body REDD1 deletion prevents RPE damage and photoreceptor loss in mice administered NaIO_3_ (15). Within the retina, REDD1 expression is dominant in Müller glia, where it acts to promote Müller glial activation, neurodegeneration, and visual dysfunction in the context of diabetes (16). The studies here were designed to investigate the role of REDD1-dependent signaling in Müller glia remodeling in the context of dry AMD.

## Results

### REDD1 deletion prevented NaIO_3_-induced Müller cell gliosis

NaIO_3_ administration induced retinal atrophy in REDD1^+/+^ mice, characterized by a decrease in the RPE marker RPE65 and the loss of photoreceptor inner and outer segments (Figs. 1*A* and S1). Consistent with our prior report (15), retinal atrophy was reduced in REDD1^−/−^ mice following NaIO_3_ administration. As a marker of Müller cell gliosis, GFAP levels were evaluated in murine retina. When REDD1^+/+^ mice were administered NaIO_3_, *Gfap* mRNA expression was increased in retinal isolates (Fig. 1*B*). However, NaIO_3_ did not increase *Gfap* mRNA expression in the retina of REDD1^−/−^ mice. Immunofluorescence analysis of retinal cross-sections revealed intense GFAP staining throughout the retina of REDD1^+/+^ mice that received NaIO_3_ (Fig. 1*C*). In regions of retinal atrophy, Müller glial processes positive for both GFAP and vimentin displayed a horizontal orientation (Fig. 1, *D* and *E*). In regions bordering the atrophic areas of the retina in REDD1^+/+^ mice administered NaIO_3_, where the outer nuclear layer was not disorganized (Fig. S2), vimentin labeling extended along the entire length of Müller glia and was accompanied by increased GFAP immunoreactivity (Fig. 1*C*). In these non-atrophic regions, Müller glial processes positive for GFAP exhibited a predominantly vertical orientation, in contrast to their horizontal alignment within atrophic areas of the outer retina. Unlike REDD1^+/+^ mice, REDD1^−/−^ mice did not exhibit NaIO_3_-induced Müller glial activation.

**Figure 1:**
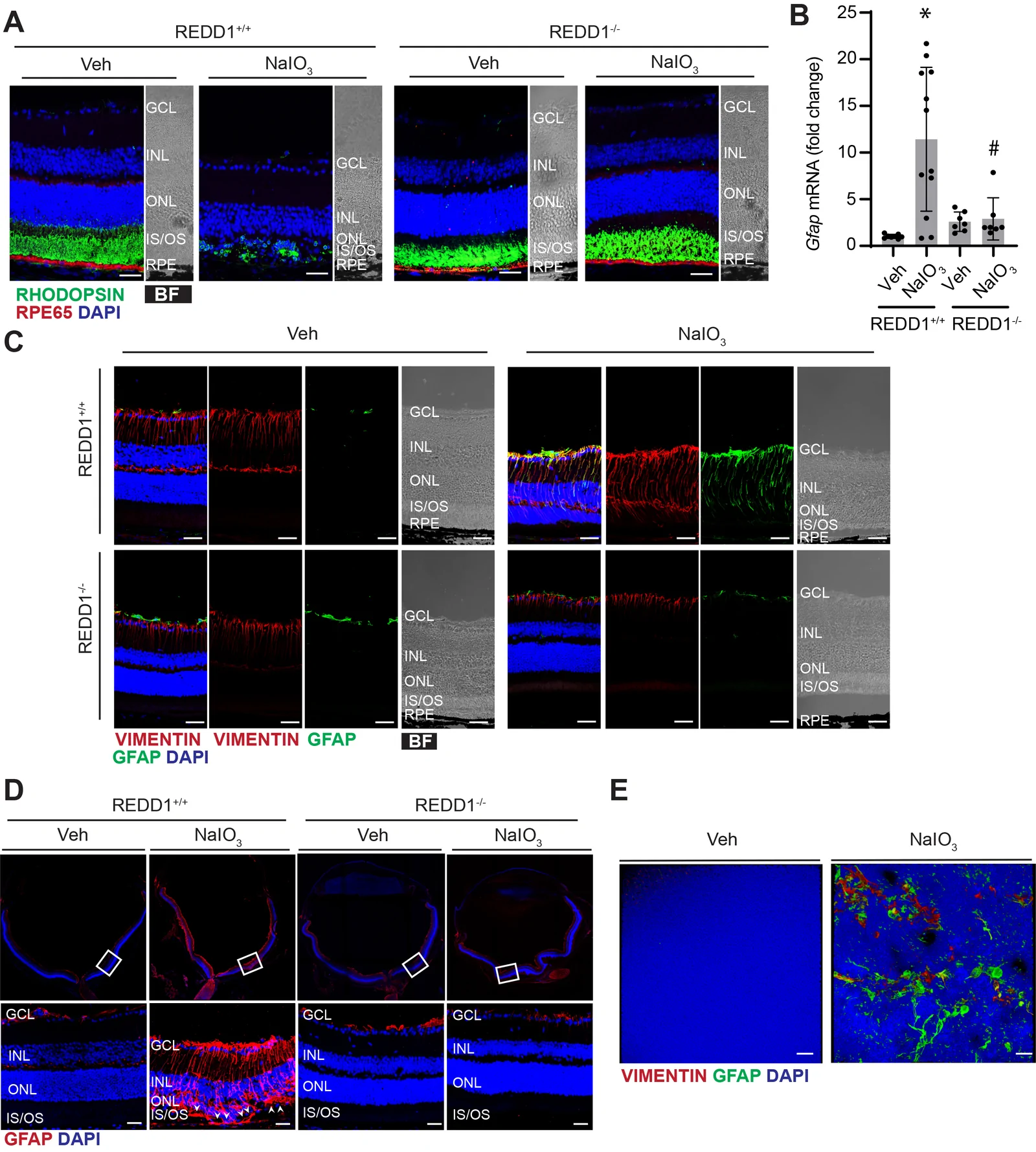
**REDD1 deletion prevented Müller glial activation after NaIO_3_ administration.** REDD1^+/+^ and REDD1^−/−^ mice were administered NaIO_3_ or saline vehicle (Veh) and retinas were analyzed 7 days later. *A*, RPE65 and rhodopsin were visualized in retinal sections by immunofluorescent microscopy. Brightfield (BF) image is shown on the *right*. Representative image panels in *A* are also shown in Figure S1, which includes individual channels for improved visualization. *B*, *Gfap* mRNA expression was quantified in retinal homogenates by RT-PCR. *C*, immunofluorescence was used to visualize vimentin and GFAP in retinal sections. Nuclei were stained with DAPI. *D*, GFAP immunostaining was used to visualize gliotic Müller glial processes. Box (*white*) indicates area shown below at increased magnification. *Arrowheads* indicate horizontal orientation of Müller glia in areas of retinal damage. Some panels in (*D*) are also shown in Figure 2*G* with addition of N-cadherin. *E*, wholemount retinas from REDD1^+/+^ mice were stained for vimentin and GFAP and imaged with the outer limiting membrane *en face*. Scale bar: 25 μm. Statistical significance was determined by one-way ANOVA followed by Tukey test for multiple comparisons. Individual data points represent independent biological replicates. ∗, *p* ≤ 0.05 *versus* Veh; #, *p* ≤ 0.05 *versus* REDD1^+/+^; GCL, ganglion cell layer; INL, inner nuclear layer; ONL, outer nuclear layer; IS/OS, photoreceptor inner segments/outer segments; RPE, retinal pigment epithelium.

### REDD1 was required for NaIO_3_-induced upregulation of mesenchymal markers in the retina

TGF-β signaling is an important driver of mesenchymal activation in Müller glia (17). Both *Tgfb1* mRNA expression and TGF-β1 protein levels were increased in the retina of REDD1^+/+^ mice after NaIO_3_ administration (Fig. 2, *A* and *B*). While there was a trend toward a modest increase in TGF-β1 levels in REDD1^−/−^ mice after NaIO_3_ administration (*p* = 0.54), TGF-β1 levels were reduced in the retina of REDD1^−/−^ mice as compared to REDD1^+/+^ mice after NaIO_3_ administration. NaIO_3_ also promoted expression of the mesenchymal markers *Cdh2* and *Vim* in the retina of REDD1^+/+^ mice (Fig. 2, *C* and *D*). Additionally, expression of *Snai1*, a key transcription factor downstream of TGF-β1 signaling and inducer of mesenchymal characteristics, was also increased in the retina of REDD1^+/+^ mice after NaIO_3_ administration (Fig. 2*E*). However, NaIO_3_ did not increase the expression of these mesenchymal markers in the retina of REDD1^−/−^ mice. Immunofluorescence staining revealed colocalization of the Müller glia marker glutamine synthetase (GS) with the mesenchymal marker N-cadherin (protein encoded by *Cdh2,* Fig. 2*F*). Furthermore, Müller glial processes positive for GFAP co-expressed N-cadherin, particularly in the outer retina of atrophic regions (Fig. 2*G*). While some Müller glia were immunoreactive for GFAP alone, a substantial proportion exhibited dual GFAP and N-cadherin labeling.

**Figure 2:**
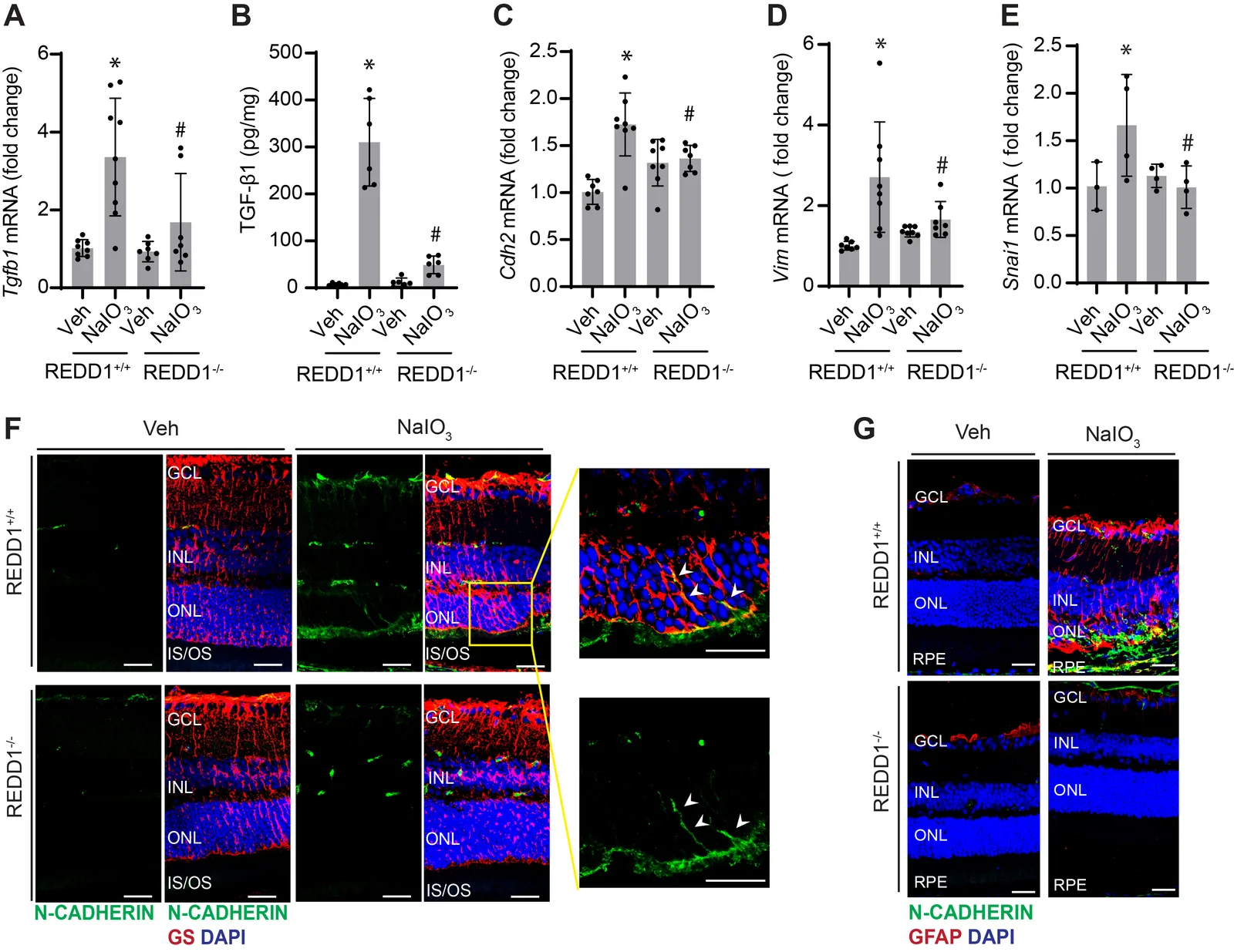
**REDD1 was required for increased mesenchymal marker expression in the retina.** REDD1^+/+^ and REDD1^−/−^ mice were administered NaIO_3_ or saline vehicle (Veh). *A*, retinal lysates were collected 7 days post-injection and *Tgfb1* mRNA expression was quantified by RT-PCR. *B*, TGF-β1 protein was measured in retinal lysates by ELISA. *C-E*, *Cdh2* (*C*), *Vim* (*D*), and *Snai1* (*E*) mRNA expression was quantified in retinal lysates by RT-PCR. *F*, retinal sections were immunolabeled for glutamine synthetase (GS) and N-cadherin. Nuclei were visualized with DAPI. Box (*yellow*) indicates area from REDD1^+/+^ mouse administered NaIO_3_ shown at *right* at increased magnification. *Arrowheads* (*white*) indicate areas with N-cadherin and GS colocalization. *G*, colocalization of GFAP and N-cadherin was visualized by immunofluorescence. Some panels in *G* are also shown in Figure 1*D* without the inclusion of N-cadherin. Scale bar: 25 μm. Statistical significance was determined by one-way ANOVA followed by Tukey test for multiple comparisons. Individual data points represent independent biological replicates. ∗, *p* ≤ 0.05 *versus* Veh; #, *p* ≤ 0.05 *versus* REDD1^+/+^; GCL, ganglion cell layer; INL, inner nuclear layer; ONL, outer nuclear layer; IS/OS, photoreceptor inner segments/outer segments; RPE, retinal pigment epithelium.

### REDD1 deletion reduced gliosis and mesenchymal marker expression in Müller cell cultures

To further explore the role of REDD1 in regulating mesenchymal marker expression in Müller cells, TGF-β signaling was investigated in wild-type and REDD1-deficient human MIO-M1 Müller cells. Due to the presence of TGF-β in the serum that was used to supplement culture media, cells were briefly cultured in serum-free media followed by TGF-β1 supplementation. TGF-β1 increased REDD1 protein and mRNA expression in Müller cells (Fig. 3, *A* and *B*). TGF-β1 also increased *GFAP* mRNA expression in wild-type cells (Fig. 3*C*), which was accompanied by a corresponding increase in GFAP protein (Fig. 3*D*). In contrast with wild-type cells, REDD1-deficient Müller cells did not exhibit a TGF-β1-induced increase in GFAP levels. Notably, *GFAP* mRNA expression following serum deprivation was also reduced in REDD1-deficient cells as compared with wild-type, suggesting an underlying role for REDD1 in Müller cell gliosis. Coincident with the reduction in gliosis, the increase in mesenchymal markers *ACTA2, SNAI1,* and *CDH2* mRNA expression in response to TGF-β1 was attenuated in REDD1-deficient Müller cells as compared to wild-type (Fig. 4, *A*–*C*). Consistent with the changes in gene expression, REDD1 deficiency also reduced the abundance of SNAI1 and αSMA (encoded by *ACTA2* mRNA) proteins in Müller cells exposed to TGF-β1 (Fig. 4, *D* and *E*), and reduced the number of αSMA-positive cells (Fig. 4, *F* and *G*). The data are consistent with a critical role for REDD1 in TGF-β1-induced mesenchymal gene expression in Müller cells.

**Figure 3:**
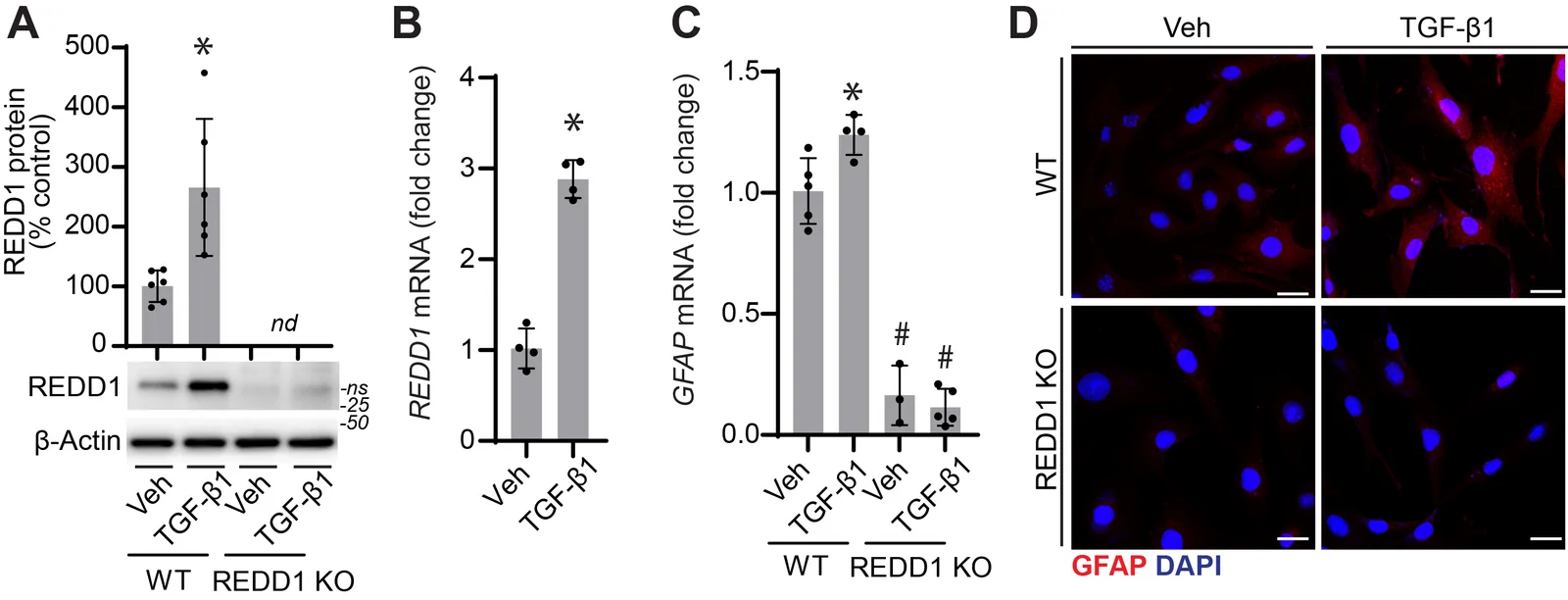
**REDD1 deletion suppressed gliosis in human Müller cell cultures exposed to TGF-β1.** Wild-type (WT) and REDD1 knockout (REDD1 KO) human MIO-M1 Müller cells were cultured for 4 h in serum-free media and then exposed to media supplemented with TGF-β1 (10 ng/ml). *A*, REDD1 protein was evaluated in cell lysates 1 h after exposure to TGF-β1 by western blotting. Representative blots are shown. Molecular mass in kDa is shown at *right* of each blot. *B, REDD1* mRNA expression was evaluated in cell lysates 1 h after exposure to TGF-β1 by RT-PCR. *C, Gfap* mRNA expression was quantified 48 h after exposure to TGF-β1. *D*, GFAP immunoreactivity was visualized 48 h after exposure to TGF-β1 (25 μm scale bar). Statistical significance was determined by one-way ANOVA followed by Tukey test for multiple comparisons. Individual data points represent independent biological replicates. ∗, *p* ≤ 0.05 *versus* Veh; #, *p* ≤ 0.05 *versus* WT; nd, not determined; ns, non-specific.

**Figure 4:**
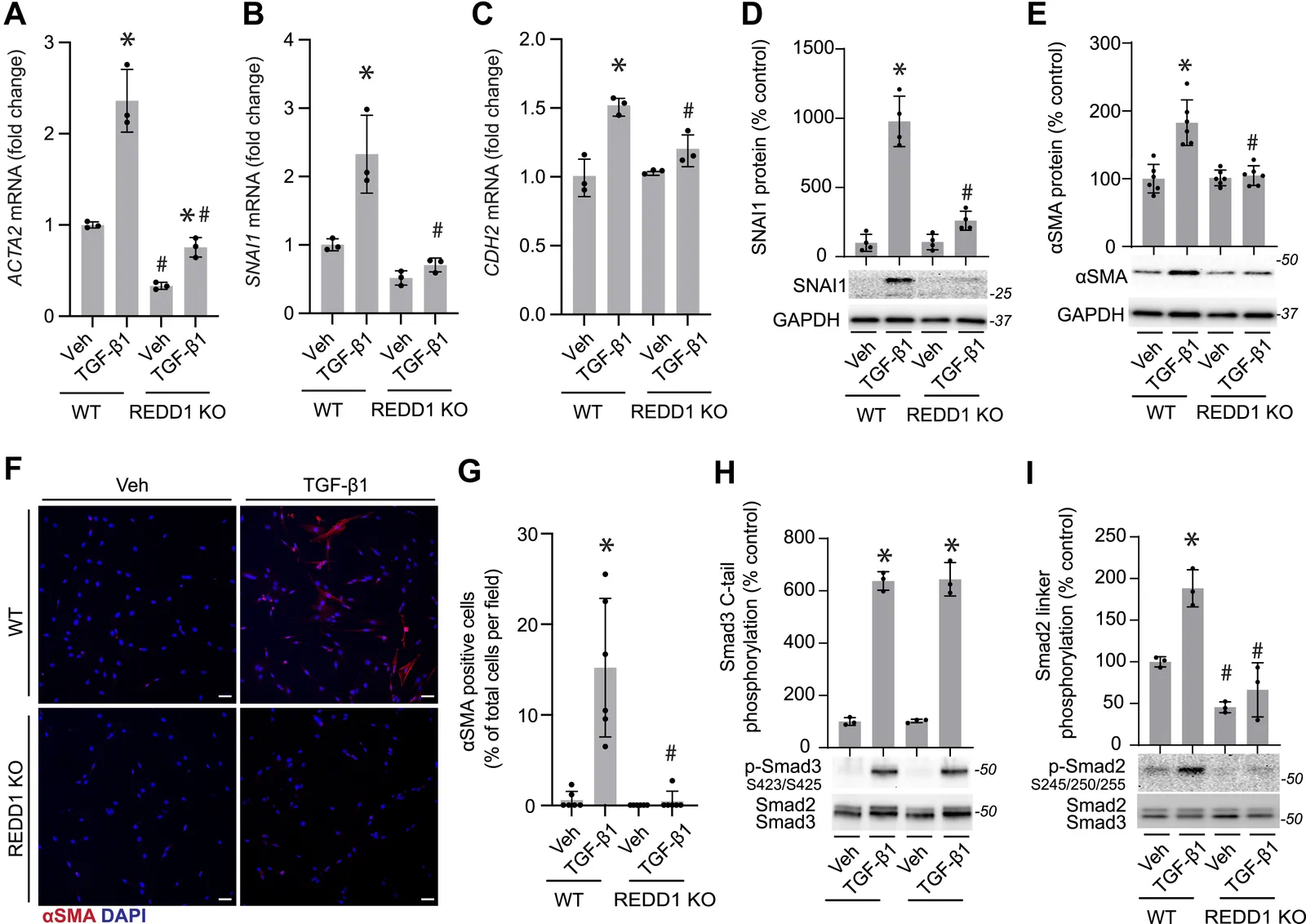
**REDD1 deletion attenuated mesenchymal gene expression in Müller cells exposed to TGF-β1.** Wild-type (WT) and REDD1 knockout (REDD1 KO) human MIO-M1 Müller cells were cultured for 4 h in serum-free media and then exposed to media supplemented with TGF-β1 (10 ng/ml) or a vehicle (Veh) control for 48 h. *A-C*, Expression of *ACTA2* (*A*), *SNAI1* (*B*), and *CDH2* (*C*) was quantified by RT-PCR. *D-E*, SNAI1 (*D*) and αSMA (*E*) protein levels were determined by Western blot analysis. *F*, αSMA (*red*) was visualized by immunofluorescence (50 μm scale bar). Nuclei were stained with DAPI (*blue*). *G,* percentage of αSMA-positive cells from *F* was quantified. *H-I*, phosphorylation of Smad3 at S423/S425 (*H*) and Smad2 phosphorylation at S245/S250/S255 (*I*) was determined by western blotting 1 h after TGF-β1 or Veh. Statistical significance was determined by one-way ANOVA followed by Tukey test for multiple comparisons. Individual data points represent independent biological replicates. ∗, *p* ≤ 0.05 *versus* Veh; #, *p* ≤ 0.05 *versus* WT.

### REDD1 enhanced SMAD linker domain phosphorylation

In Müller cells exposed to TGF-β1, canonical phosphorylation of the Smad3 C-terminal tail at S423/425 was similarly enhanced in wild-type and REDD1-deficient cells (Fig. 4*H*). While C-terminal tail phosphorylation is necessary to activate mesenchymal gene expression in response to TGF-β1, non-canonical phosphorylation of the Smad2/3 linker regions serves as an additional regulatory modification that influences SMAD2/3 transcriptional activity (18). TGF-β1 enhanced Smad2 linker phosphorylation at S245/250/255 in wild-type cells (Fig. 4*I*). Smad2 linker phosphorylation was reduced in REDD1-deficient Müller cells as compared to wild-type and REDD1 was required for TGF-β1-induced phosphorylation of the Smad2 linker. The observation supports that REDD1 influences non-canonical phosphorylation of the Smad2/3 linker domain.

### GSK3β was required for TGF-β1-induced mesenchymal markers expression in Müller cells

We previously demonstrated that REDD1 promotes GSK3β activation in Müller cells (19, 20). A prior study supports that GSK3 regulates phosphorylation of the Smad2 linker domain (21). To evaluate the role of GSK3β in mesenchymal gene expression, Müller cells were exposed to the GSK3 inhibitor VP3.15. VP3.15 suppressed the TGF-β1-induced increase in *ACTA2* and *SNAI1* mRNA expression (Fig. 5, *A* and *B*), as well as the increase in αSMA protein abundance (Fig. 5*C*). To evaluate the role of GSK3β specifically, GSK3β was knocked down by shRNA expression (Fig. 5*D*). Similar to pharmacological inhibition, GSK3β knockdown attenuated *ACTA2* and *SNAI1* mRNA expression and reduced αSMA protein abundance in Müller cells exposed to TGF-β1 (Fig. 5, *E* and *F*). GSK3β knockdown decreased the number of αSMA-positive cells (Fig. 5, *G* and *H*). Moreover, GSK3β knockdown was sufficient to attenuate TGF-β1-induced phosphorylation of the Smad2 linker domain (Fig. 5*I*). The data support a working model wherein REDD1-dependent GSK3β signaling promotes SMAD complex activation and mesenchymal gene expression in Müller cells (Fig. 5*J*).

**Figure 5:**
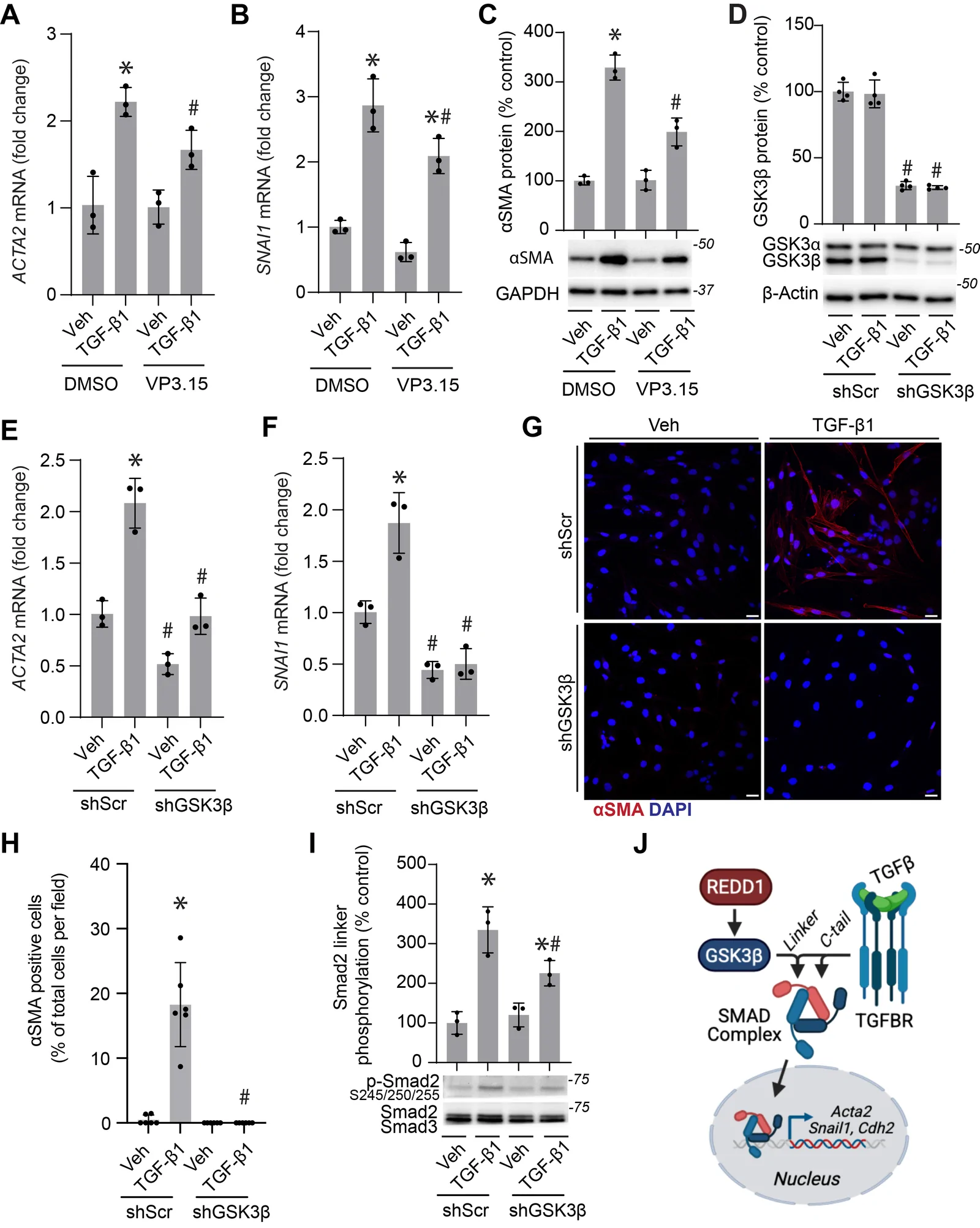
**GSK3β was necessary for increased mesenchymal marker expression in Müller cells exposed to TGF-β1.** Human MIO-M1 Müller cells were cultured for 4 h in serum-free media and then exposed to media supplemented with TGF-β1 (10 ng/ml) or a vehicle (Veh) control. *A–C*, expression of *ACTA2* (*A*) and *SNAI1* (*B*) mRNA in cells exposed to TGF-β1 for 48 h in the presence of either the GSK3 inhibitor VP3.15 (1 μm) or DMSO vehicle was quantified by RT-PCR, and αSMA (*C*) protein levels were determined by Western blotting. *D–I*, GSK3β was knocked down by stable expression of an shRNA (shGSK3β). Control cells expressed a scramble shRNA (shScr). Western blotting was used to assess GSK3β knockdown after 1h of TGF-β1 or Veh (*D*). Expression of *ACTA2* (*E*) and *SNAI1* (*F*) was quantified by RT-PCR. αSMA (*red*) was visualized by immunofluorescence (25 μm scale bar) 48 h after TGF-β1 or Veh exposure (*G*). Nuclei were stained with DAPI (*blue*). *H,* percentage of cells positive for αSMA in (*G*) was quantified. *I*, Smad2 phosphorylation at S245/250/255 was determined by western blotting after 1h of TGF-β1 or Veh. Statistical significance was determined by one-way ANOVA followed by Tukey test for multiple comparisons. Individual data points represent independent biological replicates. ∗, *p* ≤ 0.05 *versus* Veh; #, *p* ≤ 0.05 *versus* WT, DMSO, or shScr. *J*, working model for REDD1-dependent activation of GSK3β leading to enhanced SMAD complex activation and mesenchymal gene expression in Müller glia.

### Müller glia-specific REDD1 deletion failed to prevent NaIO_3_-induced retinal damage

To investigate the role of REDD1-dependent Müller cell reprogramming in retinal degeneration, cre-lox recombination was used to specifically delete REDD1 in Müller glia (16). As previously reported (16), REDD1 expression was dramatically reduced in the retina of mice with Müller glia-specific REDD1 deletion (REDD1 mgKO, Fig. 6*A*). NaIO_3_ promoted retinal gliosis in REDD1^fl/fl^ mice, which was evidenced by increased GFAP staining that colocalized with vimentin (Fig. 6, *B* and *C*). NaIO_3_-induced GFAP staining was reduced in the retina of REDD1 mgKO mice as compared to REDD1^fl/fl^ mice, and the horizontal orientation of GFAP-positive Müller glial processes within the subretinal space was absent (white arrows in Fig. 6*C*). Despite the reduction in Müller cell reprogramming, RPE and photoreceptor damage was similar in REDD1 mgKO mice and floxed control mice after NaIO_3_ administration (Fig. 6, *D* and *E*). To determine if the REDD1-dependent change in Müller glial activation was associated with improved visual function, thresholds for the virtual optomotor response were determined 3 days after NaIO_3_ administration. Spatial frequency threshold and contrast sensitivity were only modestly improved in REDD1 mgKO mice compared with REDD1^fl/fl^ controls after NaIO_3_ administration (Fig. 6, *F* and *G*). The findings support the role of REDD1-dependent signaling in Müller cell reprogramming and highlight a critical role for REDD1 in cells beyond Müller glia in NaIO_3_-induced retinal damage.

**Figure 6:**
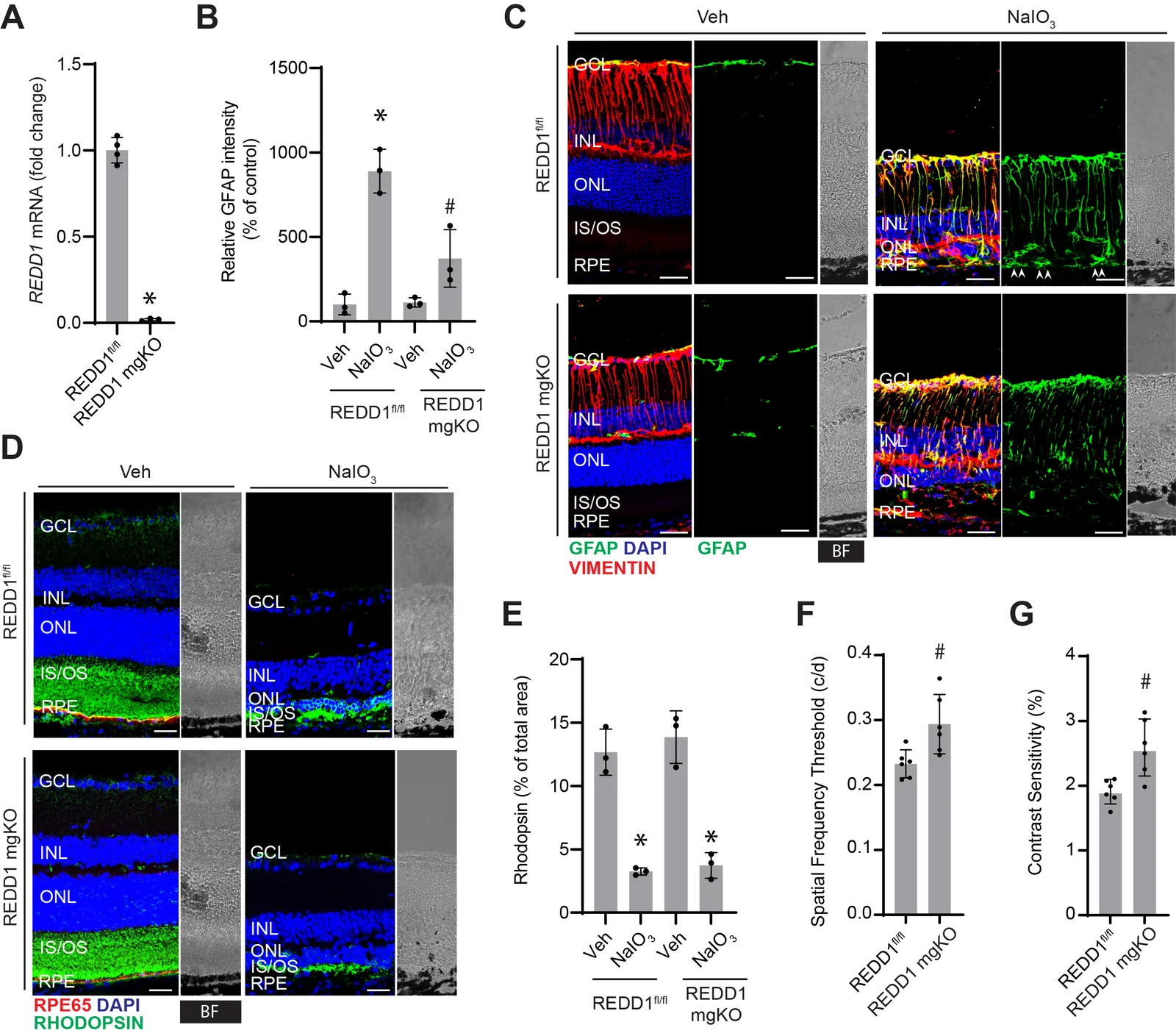
**Müller glia-specific REDD1 deletion attenuated gliosis despite persistent NaIO_3_-induced retinal damage.** REDD1^fl/fl^ and REDD1 mgKO mice were administered NaIO_3,_ or saline vehicle (Veh) and eyes were collected after 7 days. *A*, REDD1 mRNA expression was quantified in retinal lysates by RT-PCR. *B–C*, Vimentin and GFAP were visualized in retinal sections by immunofluorescence to evaluate Müller glia activation. Nuclei were stained with DAPI. Corresponding brightfield (BF) images are shown on the *right*. *Arrowheads* (*white*) indicate horizontal orientation of Müller glia. Relative GFAP intensity was quantified using ImageJ. *D–E*, RPE65 and rhodopsin were visualized in retinal sections by immunofluorescent microscopy to evaluate retinal damage. The rhodopsin-positive area was quantified using ImageJ. *F–G*, visual function was evaluated 3 days after NaIO_3_ administration. Spatial frequency threshold was assessed at 100% contrast. Contrast sensitivity was assessed at a spatial frequency of 0.092 cycles/degree (c/d). Scale bar: 25 μm. Statistical significance (*B* and *E*) was determined by one-way ANOVA followed by Tukey test for multiple comparisons. Data in (*A* & *F*–*G*) were analyzed by unpaired Student’s *t* test. Individual data points represent independent biological replicates. ∗, *p* ≤ 0.05 *versus* REDD1^fl/fl^ or Veh; GCL, ganglion cell layer; INL, inner nuclear layer; ONL, outer nuclear layer; IS/OS, photoreceptor inner segments/outer segments; RPE, retinal pigment epithelium.

## Discussion

We previously demonstrated that NaIO_3_ promotes REDD1 protein abundance in both the retina and the RPE of mice, and that whole-body REDD1 deletion is sufficient to protect mice from NaIO_3_-induced retinal degeneration (15). Herein, whole-body or Müller cell–specific REDD1 deletion reduced NaIO_3_-induced Müller cell gliosis and morphological remodeling. Consistent with these observations in murine retina, REDD1 was an essential mediator of gliosis and increased mesenchymal markers in human Müller cell cultures exposed to TGF-β1. The data provide evidence that REDD1 promotes TGF-β1-induced Müller glial remodeling through a mechanism involving GSK3β-dependent SMAD complex phosphorylation. Importantly, preservation of the outer retina and the lack of gliosis in whole-body REDD1 knockout mice administered NaIO_3_ was not exclusively due to the absence of REDD1 in Müller glia, because Müller glia-specific REDD1 knockout mice did not exhibit retinal protection that was similar to whole-body REDD1 knockout mice. The observation implies a critical role for REDD1 in cells other than Müller glia (*e.g.*, RPE/photoreceptors) in NaIO_3_-induced retinal degeneration. Thus, REDD1 potentially acts independently to drive both retinal atrophy and Müller glia reprogramming in the context of dry AMD.

Dry AMD is characterized by primary damage to the RPE and the secondary loss of photoreceptors. The oxidant stressor NaIO_3_ recapitulates features of dry AMD in laboratory animals, including RPE atrophy, photoreceptor degeneration, and retinal inflammation (22, 23). RPE are the primary target of NaIO_3_-induced damage, where it induces oxidative stress, mitochondrial dysfunction, and cell death, potentially reflecting a high affinity of the compound for this cell type (24, 25). RPE provides essential metabolic and structural support to the photoreceptors, including nutrient transport, phagocytosis of shed outer segments, and regeneration of visual pigment; thus, RPE injury leads to secondary photoreceptor degeneration and generates a pattern of regional retinal atrophy that models GA (26). NaIO_3_ administration is known to produce variable degrees of retinal damage across animals (26), and we observed similar inter-group variability among REDD1^+/+^ mice in this study. In contrast, REDD1^−/−^ mice were protected from NaIO_3_-induced retinal damage and exhibited more consistent outcomes.

In addition to RPE damage, it is well established that NaIO_3_ causes retinal Müller cell gliosis (27, 28, 29). In healthy retina, expression of the gliosis marker GFAP is restricted to astrocytes within the inner retina, while Müller glia exhibit minimal GFAP expression. Following NaIO_3_-induced injury, Müller glia display marked GFAP upregulation, reflecting gliosis (27). In response to NaIO_3_ administration, Müller cell gliosis was pronounced in wild-type mice, but not in REDD1-deficient mice. The absence of retinal injury in whole-body REDD1 knockout mice likely contributes to the reduction in Müller glial activation, independently of REDD1 signaling within Müller cells, as there is a reduced stimulus for the response. In addition to gliosis, NaIO_3_-induced retinal injury was characterized by substantial remodeling of Müller glial processes within atrophic regions of the retina. The observation is consistent with the prior report that, in human retinas with GA, Müller glial processes exhibit a horizontal orientation within the subretinal space (4, 30). This Müller glial reorganization may represent an adaptive response to RPE loss and disruption of the outer blood-retinal barrier through Müller cell migration and the formation of a subretinal glial membrane. Similar subretinal glial membranes have also been observed in rodents with NaIO_3_-induced retinal injury (4). Notably, REDD1 expression specifically in Müller glia was required for these morphological changes, supporting a key role for REDD1 in maladaptive glial remodeling. The mesenchymal marker N-cadherin was upregulated in the outer retina after NaIO_3_-induced retinal injury; however, a similar response was not observed with the mesenchymal marker αSMA (Fig. S3, *A* and *B*). Thus, the NaIO_3_-induced Müller glia remodeling described herein likely represents partial glial-to-mesenchymal transition, rather than a complete phenotype shift reported in other models. It is also important to acknowledge that the co-localization of N-cadherin with GS and GFAP in retinal cross-sections could also be influenced by expression in other cells within the sub-retinal space.

We previously discovered a protective effect of Müller glia-specific REDD1 deletion in visual function deficits in diabetic mice (16). Herein, Müller glia-specific deletion of REDD1 only modestly preserved visual function in the context of NaIO_3_-induced retinal injury. NaIO_3_ induces RPE damage and secondary photoreceptor degeneration, which are major contributors to visual dysfunction (31). Although Müller glia are not directly involved in phototransduction, they maintain retinal structural integrity and are essential in preserving photoreceptor function through the regulation of retinal metabolism, potassium and water homeostasis, glutamate recycling, antioxidant defenses, and trophic factor signaling (32). Indeed, Müller glia can partially compensate for the impact of RPE deficits on photoreceptors in retinal disease such as retinitis pigmentosa (33). Müller cell gliosis and remodeling are accompanied by loss of normal Müller cell properties that lead to changes in the retinal microenvironment, glial scar formation, and disrupted outer retinal architecture, thereby leading to photoreceptor loss and deficits in synaptic transmission. Thus, the modest improvement in visual function in mice with Müller glia-specific REDD1 deletion after NaIO_3_ administration likely reflects Müller glial support that maintains function of surviving photoreceptors.

Beyond the gliosis and remodeling observed in activated Müller glia, they also secrete pro-inflammatory and trophic factors that drive retinal disease progression. Specifically, TGF-β is a well-established mediator of fibrotic retinal disease (34), which is increased in the eyes of patients with neovascular AMD (35). Consistently, increased TGF-β1 expression and signaling have been reported in the retinas of mice following NaIO_3_ administration (36). Herein, NaIO_3_ administration increased TGF-β1 levels in the retina in a REDD1-dependent manner. TGF-β1 promotes gliosis and negatively influences Müller glial homeostatic functions, whereas inhibition of TGF-β signaling attenuates gliosis in rats subjected to light-induced retinal injury (37). In addition to promoting gliosis, TGF-β1 drives mesenchymal activation in Müller glia, a process increasingly recognized in retinal diseases (11). REDD1 deletion markedly attenuated the increase in mesenchymal marker expression both in the retina of mice receiving NaIO_3_ and in human Müller cell cultures exposed to TGF-β1. Although our findings support that REDD1 is required for NaIO_3_-induced TGF-β1 upregulation and mesenchymal marker expression, they do not exclude contributions from additional pro-fibrogenic signaling pathways and other retinal cell types that are responsive to NaIO_3_ administration. Moreover, the MIO-M1 cell culture model is a helpful tool for exploring cell-intrinsic mechanisms, such as TGF-β1 signaling and mesenchymal gene expression, but does not fully recapitulate the complex intercellular signaling that occurs within the retina in the context of dry AMD.

Prior studies support the role of REDD1 in fibrotic disease progression; however, its role in retinal fibrosis and glial remodeling has not been previously examined. Indeed, REDD1 was previously implicated in pro-fibrotic signaling in the context of diabetic nephropathy (38), and it was also found to contribute to TGF-β1 signaling in the context of hepatic fibrosis (39). The TGF-β1 was shown to increase REDD1 expression at the transcriptional level through the activation of MAPK/AP-1 signaling (39). The increased REDD1 levels observed in Müller cells following TGF-β1 exposure are consistent with these reports, suggesting that a similar transcriptional mechanism may underlie REDD1 induction in Müller glia. The data here further support that REDD1 is required for upregulation of SNAI1, which serves as a key regulator of mesenchymal activation in Müller glia (12). In the retinas of mice receiving NaIO_3_ and in human Müller cell cultures exposed to TGF-β1, REDD1 deletion prevented an increase in SNAI1. Prior studies support that SNAI1 is required for TGF-β1-induced loss of Müller cell properties, as it contributes to Müller cell gliosis and mesenchymal marker expression in response to diabetic conditions (11, 12). The studies here identify REDD1 as a critical regulator of SNAI1 in Müller glia.

Canonical TGF-β signaling involves phosphorylation of Smad2/3 at C-terminal serine residues by the TGF-β receptor. Canonical phosphorylation of Smad3 at S423/S425 within the C-terminal tail was enhanced in Müller cells exposed TGF-β1 and REDD1 deletion did not alter Smad3 C-terminal phosphorylation. TGF-β-mediated phosphorylation of Smad2/3 promotes formation of a heteromeric complex with Smad4 and translocation into the nucleus. While Smad2/3 C-terminal tail phosphorylation is necessary for increased mesenchymal gene expression, it is not sufficient (18). Indeed, Smad2/3 linker phosphorylation regulates fibrotic signaling in multiple tissues (40, 41) and acts as a critical regulator of Smad-dependent transcriptional activity (42). REDD1 deficiency prevented TGF-β1-induced phosphorylation of the Smad2 linker domain at S245/S250/S255 and blocked the induction of mesenchymal genes. Consistent with this observation, a previous study demonstrated that inhibiting Smad2 linker phosphorylation at S245/S250/S255 reduced TGF-β1-induced collagen 1 upregulation in fibroblasts, despite having no effect on Smad2 C-terminal phosphorylation (43).

Previous studies also support a key role for GSK3β in regulating noncanonical Smad signaling, including Smad2 linker phosphorylation at S245/250/255 (21). REDD1 acts by recruiting protein phosphatase 2A to Akt, promoting site-specific dephosphorylation of Akt at T308 and thereby reducing Akt kinase activity (44). Reduced Akt activity decreases the inhibitory phosphorylation of GSK3β at S9, resulting in enhanced GSK3β activity (45). Consistent with this mechanism, REDD1-deficient Müller cells exhibit increased inhibitory phosphorylation of GSK3β at S9, and reduced phosphorylation of its best-known target glycogen synthase (19, 46). Herein, GSK3β knockdown attenuated TGF-β1-induced mesenchymal marker expression and suppressed Smad2 linker phosphorylation. Together, these findings support a model in which REDD1 enhances TGF-β1-induced mesenchymal marker expression by enhancing Smad2 linker phosphorylation through a GSK3β-dependent signaling axis.

Müller glia undergo profound structural and functional changes in the progression of GA (2, 3, 4, 8, 47). While Müller glia possess regenerative capacity in response to retinal injury in some vertebrate species, this capacity is absent in mammals (48). In mammals, glial injury triggers reactive gliosis, remodeling, and ultimately leads to irreversible degeneration. Activation of the TGF-β signaling pathway is a key driver of Müller glial activation and remodeling in the mammalian retina; however, the precise molecular mechanisms that regulate TGF-β signaling in this context are poorly understood. Our previous studies demonstrated that REDD1 contributes to diabetes-induced Müller glial activation, and the current findings extend these observations by identifying the role of REDD1 in Müller glial activation and Müller cell remodeling after NaIO_3_-induced retinal damage. The findings support Müller glial REDD1 as a critical link between retinal injury and maladaptive glial reprogramming. However, deletion of REDD1 specifically in Müller glia was insufficient to recapitulate the protection from NaIO_3_-induced retinal damage observed with germline REDD1 deletion (15), implying a critical role for REDD1 in cells beyond Müller glia in pathological retinal degeneration.

## Experimental procedures

### Animals

All animal procedures were approved by the Institutional Animal Care and Use Committee of the Penn State College of Medicine and were conducted in accordance with the ARVO Statement for the Use of Animals in Ophthalmic and Vision Research. Eight-week-old B6;129 REDD1^+/+^ and REDD1^−/−^ mice (49) were maintained under 12-h light/dark cycle with ad libitum access to food and water. Mice were randomly assigned to experimental groups, and littermate controls were used whenever possible. Both male and female mice were used in this study, with approximately equal numbers distributed across experimental groups. No sex differences were observed. For low-dose NaIO_3_ administration, a sterile solution was freshly prepared in 0.9% saline, passed through a 0.22 μm filter, and administered as a single intraperitoneal injection at 20 mg/kg, as previously described (15). Control animals received equivalent volumes of saline vehicle. Müller glial-specific REDD1 knockout (REDD1 mgKO) mice were generated by crossing hemizygous C57BL/6-Tg(Pdgfra-cre)1Clc/J (Strain # 013148; The Jackson Laboratory, Bar Harbor, ME, USA) with homozygous REDD1-floxed (REDD1^fl/fl^) mice (16). Behavioral optometry was performed using an OptoMotry virtual optomotor system (Cerebral Mechanics), as previously described (16) 3 days after NaIO_3_ administration. Spatial frequency threshold was assessed at 100% contrast and contrast sensitivity was determined at a spatial frequency of 0.092 cycles/degree. Seven days after NaIO_3_ administration, whole eyes or retinas were collected for analysis. Experimental endpoints were selected based on our previous study (15).

### Müller cell culture

Human MIO-M1 Müller cells were obtained from the UCL Institute of Ophthalmology. REDD1-deficient MIO-M1 cells were generated using CRISPR-Cas9 genome editing as previously described (50). MIO-M1 cells stably expressing an shRNA targeting GSK3β were generated as previously described (19, 46). Cells were maintained in Dulbecco’s Modified Eagle’s Medium (DMEM; Thermo Fisher Scientific) containing 5.6 mM glucose and supplemented with 10% heat-inactivated fetal bovine serum and 1% penicillin-streptomycin. All cell cultures were monitored for *mycoplasma* contamination. To induce mesenchymal activation, cells were exposed to serum-deficient medium for 4 h and then the medium was supplemented with recombinant human transforming growth factor-β1 (TGF-β1; 10 ng/ml; PeproTech) or vehicle.

### Quantitative real-time PCR

Total RNA was isolated with TRIzol reagent (Invitrogen) according to the manufacturer’s instructions. 1 μg of total RNA was reverse-transcribed using the High-Capacity cDNA Reverse Transcription Kit (Applied Biosystems). Quantitative real-time PCR was performed using QuantiTect SYBR Green Master Mix (Qiagen) on a QuantStudio 12K Flex Real-Time PCR System (Thermo Fisher Scientific; RRID: SCR_021098). Primer sequences are listed in Supporting Information (Table S1). Mean cycle threshold (CT) values were calculated for control and experimental samples, and relative mRNA expression was determined using the 2^−^^ΔΔCT^ method, with GAPDH serving as the internal reference gene.

### Protein analysis

Retinas were collected for protein extraction as previously described (15). Protein concentrations were determined using the DC protein assay (Bio-Rad) following the manufacturer’s instructions. Retinal homogenates or cell lysates were mixed with Laemmli buffer, boiled, and separated on Criterion TGX Precast 4 to 20% gradient gels (Bio-Rad). Proteins were transferred onto polyvinylidene fluoride (PVDF) membranes. Membranes were blocked with 5% nonfat milk prepared in Tris-buffered saline containing Tween-20 (TBST) and subsequently incubated with the appropriate primary and secondary antibodies (Supporting Information- Table S2). Protein bands were visualized using the Clarity ECL detection reagent (Bio-Rad) and a FluorChem E imaging system (ProteinSimple). Densitometric analysis was performed with ImageJ software, and protein levels were normalized to GAPDH or β-actin as internal loading controls. TGF-β1 levels were determined in retinal lysates using the DuoSet mouse ELISA kit (DY240-05; R&D Systems).

### Immunofluorescent microscopy

Whole eyes were fixed in 4% paraformaldehyde (PFA) for 30 min, washed with 1X PBS and stored in 30% sucrose containing 0.05% sodium azide at 4 °C. Eyes were then embedded in optimal cutting temperature compound, flash frozen, and sectioned at 10 μm thickness using a cryostat. Retinal sections were fixed in 4% PFA for 10 min and permeabilized in PBS with 0.1% Triton X-100 (PBST). For cell staining, MIO-M1 cells were seeded on chamber slides (CELLTREAT Scientific Products) and cultured overnight prior to TGF-β1 treatment. Cells were fixed in 4% PFA and permeabilized with PBST. αSMA-positive cells were counted by an observer masked to sample identity and the values on 6 fields from two independent experiments. The GFAP fluorescence intensities were quantified using ImageJ software with a constant threshold setting to eliminate background. Tissue sections or cells were blocked with 10% normal donkey serum for 1 h at room temperature and incubated with primary antibodies followed by fluorophore-conjugated secondary antibodies (Table S2). As a negative control, sections were also processed with only secondary antibody (Fig. S3*C*). For consistency, retinal cross-section images from all experimental groups were acquired at equivalent distances from the optic nerve. For retinal flat-mount immunostaining, whole eyes were fixed in 4% PFA, and the cornea and lens were removed as previously described (15). The neuroretina was carefully dissected with four radial incisions, rinsed three times in PBS for 5 min, and blocked in 5% donkey serum for 1 h at room temperature prior to incubation with primary and secondary antibodies. Flat-mount images were acquired using a Leica SP8 FALCON confocal microscope by capturing z-stacks spanning from the ganglion cell layer to the outer limiting membrane.

### Statistical analysis

Results are expressed as mean ± SD. GraphPad Prism software was used to determine statistically significant differences in groups by Student *t* test or one-way ANOVA (*p* value < 0.05) followed by Tukey test for multiple comparisons. Statistical outliers were identified using the ROUT method in GraphPad Prism and excluded prior to analysis, with the Q value set at 1%. Trend test and pairwise comparisons were conducted with the Tukey test for multiple comparisons.

## Data availability

Primary data including unedited Western blot images are available at https://doi.org/10.6084/m9.figshare.32619819. Excel files with raw data supporting findings are publicly available from ScholarSphereat https://doi.org/10.26207/36r1-j.

## Supporting information

This article contains supporting information.

## Conflict of interest

The authors declare that they have no conflicts of interest with the contents of this article.
