# Supplementary material for: Regulation of Müller glial reprogramming by the stress response protein REDD1 and its limitations in preventing retinal degeneration
Source: J Biol Chem. 2026 Sep 23;302(11):113583. doi: 10.1016/j.jbc.2026.113583 (PMC13696805; doi:10.1016/j.jbc.2026.113583)
Supplement: Supplementary file 1 [file mmc1.pdf]

Supporting Information

Regulation of Müller glial reprogramming by the stress response protein REDD1 and its limitations in preventing retinal degeneration

Subrahmanian, S. M., et al

Table of Contents:

1. Table S1: Primers used for RT-PCR
2. Table S2: Antibodies used for western blotting and immunofluorescence
3. Figure S1. REDD1 deletion reduced $NaIO_3$-induced RPE and photoreceptor damage.
4. Figure S2. Identification of atrophic and bordering regions following $NaIO_3$-induced injury.
5. Figure S3. αSMA expression in the retina was not altered after $NaIO_3$ administration.

Table S1. Primers used for gene expression analysis

	Gene	Primer sequence (5'-3')	
		Forward	Reverse
Mouse	*Gfap*	GCGAAGAAAACCGCATCACC	GTGACTTTTTGGCCTTCCCC
	Tgfb1	AGCTGCGCTTGCAGAGATTA	AGCCCTGTATTCCGTCTCC
	Cdh2	AGGCTTCTGGTGAAATTGCAT	GTCCACCTTGAAATCTGCTGG
	Vim	GCGAGAGAAATTGCAGGAGGA	CGTTCAAGGTCAAGACGTGC
	Snai1	CACACGCTGCCTTGTGTCT	GGTCAGCAAAAGCACGGTT
	Redd1	GGGATCGTTTCTCGTCCTCC	ATGAGGAGTCTTCCTCCGGC
	Gapdh	GGTGGTCTCCTCTGACTTCAACA	GTTGCTGTAGCCAAATTCGTTGT
Human	*GFAP*	GCACGCAGTATGAGGCAATG	TAGTCGTTGGCTTCGTGCTT
	ACTA2	TGGCTATTCCTTCGTTACTACTGCT	CATCAGGCAACTCGTAACTCTTCTC
	CDH2	AGCCAACCTTAACTGAGGAGT	GGCAAGTTGATTGGAGGGATG
	REDD1	CTCTTCGCCCTCGTCCTTG	TCCAGGTAAGCCGTGTCTTC
	SNAI1	TCGGAAGCCTAACTACAGCGA	AGATGAGCATTGGCAGCGAG
	GAPDH	GTTGTCTCCTGCGACTTCA	TGCTGTAGCCGTATTCATTG

Table S2. Antibodies used for western blot and immunofluorescence

Antibody	Company	Catalogue	Lot	Dilution	Assay
REDD1	ProteinTech	10638-1-AP	00110532	1:1000	Western Blot
N-Cadherin	Cell Signaling	14215S	4	1:1000	Western Blot
				1:400	Immunofluorescence
αSMA	Cell Signaling	19245S	6	1:1000	Western Blot
				1:200	Immunofluorescence
SNAI1	Cell Signaling	3879S	14	1:1000	Western Blot
				1:100	Immunofluorescence
Actin	Cell Signaling	4970	19	1:1000	Western Blot
GAPDH	Santa Cruz	sc-47724	H1021	1:2000	Western Blot
GFAP	Sigma	G6171	0000397433	1:200	Immunofluorescence
GFAP	DAKO	Z0334	41765739	1:2000	Western Blot
				1:400	Immunofluorescence
GSK3α/β	Cell Signaling	5676	4	1:1000	Western Blot
Phospho Smad2 (S245/250/255)	Cell Signaling	3104	6	1:1000	Western Blot
Phospho Smad3 (S423/425)	Rockland	600-401-919	46905	1:1000	Western Blot
Smad2/3	Cell Signaling	8685	7	1:1000	Western Blot
Donkey anti-Mouse Alexafluor 488	Jackson ImmunoResearch	715-546-151	125414	1:800	Immunofluorescence
Donkey anti-Rabbit Alexafluor 594	Jackson ImmunoResearch	711-585-152	164523	1:800	Immunofluorescence

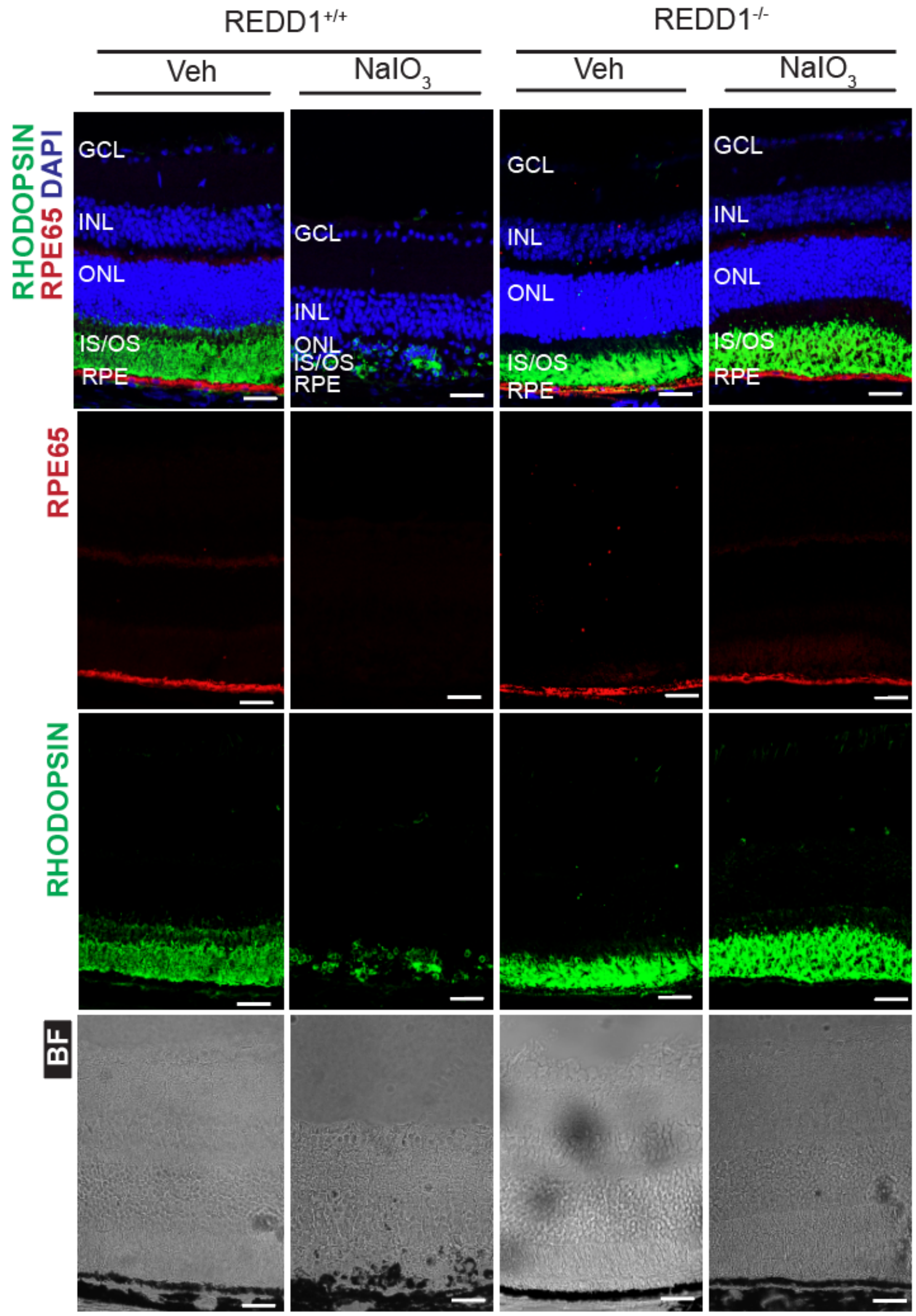

Figure S1. REDD1 deletion reduced $NaIO_3$-induced RPE and photoreceptor damage. Retinal images from Fig. 1A are shown with the inclusion of individual channel panels to improve data transparency. REDD1$^{+/+}$ and REDD1$^{-/-}$ mice were administered $NaIO_3$ or saline vehicle (Veh) and retinas were analyzed 7 days later. Immunostaining was used to visualize RPE65 (*red*) and rhodopsin (*green*) in retinal sections. Nuclei were stained with DAPI. Corresponding brightfield (BF) image is shown at the bottom. GCL, ganglion cell layer; INL, inner nuclear layer; ONL, outer nuclear layer; IS/OS, photoreceptor inner segments/outer segments; RPE, retinal pigment epithelium.

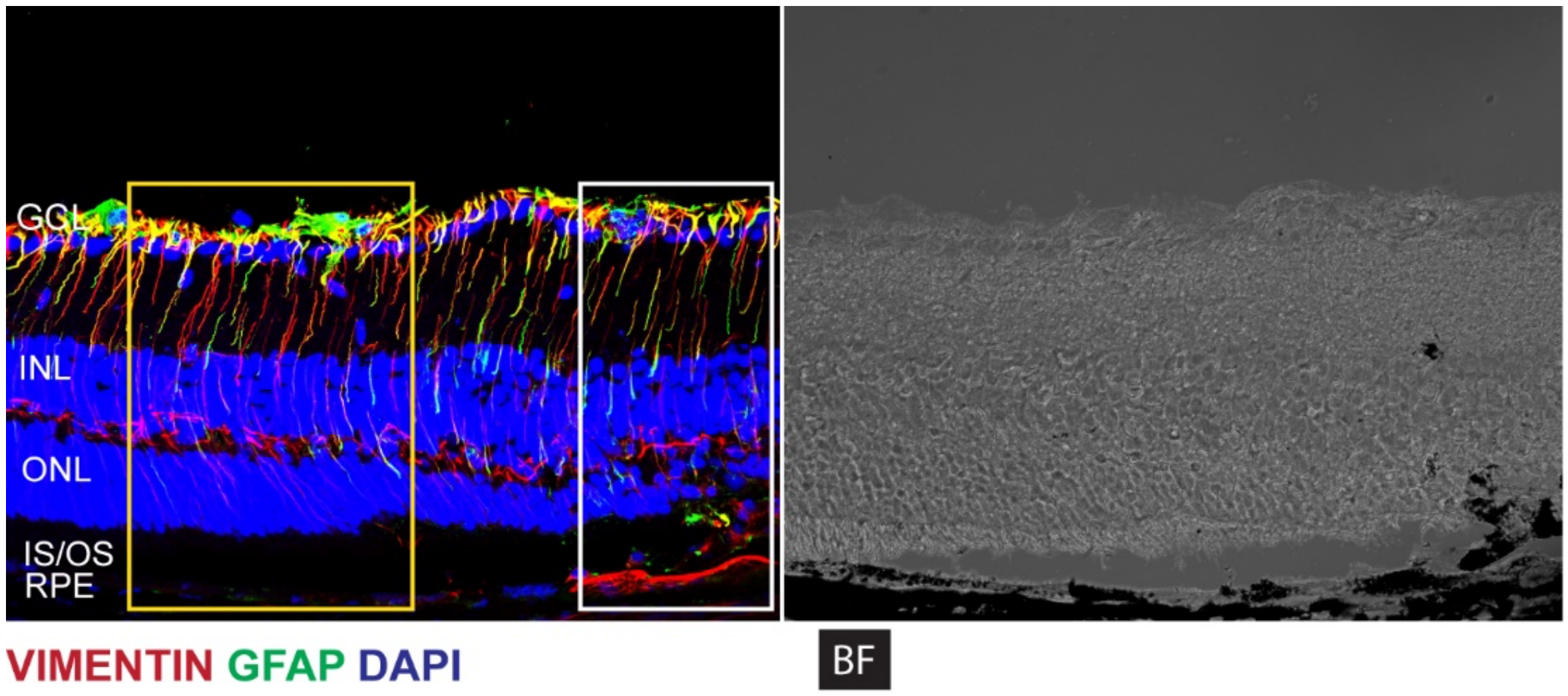

Figure S2. Identification of atrophic and bordering regions following $NaIO_3$-induced injury. Representative retinal sections from REDD1$^{+/+}$ mice 7 days after $NaIO_3$ administration were immunostained for vimentin (*red*) and GFAP (*green*) to visualize Müller glial activation. The corresponding brightfield (BF) image is shown on the right. The *white box* indicates atrophic regions and *yellow box* indicates regions adjacent to atrophic region used for analysis. GCL, ganglion cell layer; INL, inner nuclear layer; ONL, outer nuclear layer; IS/OS, photoreceptor inner segments/outer segments; RPE, retinal pigment epithelium.

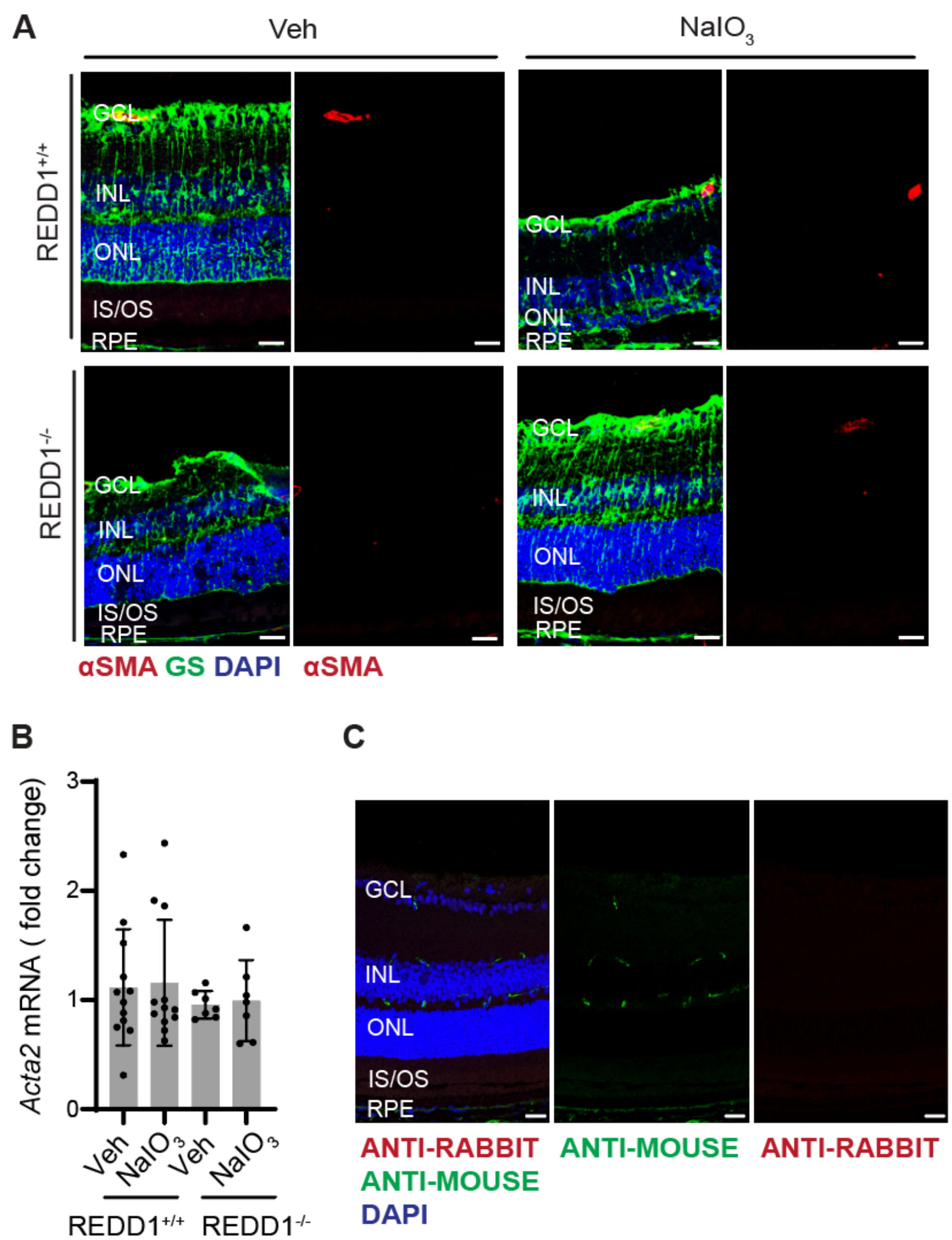

Figure S3. αSMA expression in the retina was not altered after $NaIO_3$ administration. REDD1$^{+/+}$ and REDD1$^{-/-}$ mice were administered $NaIO_3$ or saline vehicle (Veh). *A*, Representative immunofluorescence images of retinal sections from mice, 7 days after $NaIO_3$ administration, stained for αSMA (*red*) and glutamine synthetase (GS) (*green*). *B*, *Acta2* mRNA expression was evaluated in retinal homogenates by RT-PCR. *C*, Retinal sections from REDD1$^{+/+}$ mice receiving Veh were analyzed as a secondary antibody-only negative control by visualizing the background signal from donkey anti-mouse Alexa Fluor 488 (*green*) and donkey anti-rabbit Alexa Fluor 594 (*red*). Nuclei were stained with DAPI. Scale bar: 25 µM. Statistical significance was determined by one-way ANOVA followed by Tukey test for multiple comparisons. Individual data points represent independent biological replicates. GCL, ganglion cell layer; INL, inner nuclear layer; ONL, outer nuclear layer; IS/OS, photoreceptor inner segments/outer segments; RPE, retinal pigment epithelium.
